# Lipoprotein Processing and Sorting in Helicobacter pylori

**DOI:** 10.1128/mBio.00911-20

**Published:** 2020-05-19

**Authors:** Mark S. McClain, Bradley J. Voss, Timothy L. Cover

**Affiliations:** a Department of Medicine, Vanderbilt University School of Medicine, Nashville, Tennessee, USA; b Department of Pathology, Microbiology and Immunology, Vanderbilt University School of Medicine, Nashville, Tennessee, USA; c Veterans Affairs Tennessee Valley Healthcare System, Nashville, Tennessee, USA; McGovern Medical School

**Keywords:** Toll-like receptor 2, *Helicobacter pylori*, lipoproteins, posttranslational protein modification, type IV secretion systems

## Abstract

Our current understanding of lipoprotein synthesis and localization in Gram-negative bacteria is based primarily on studies of Escherichia coli. Newly synthesized E. coli prolipoproteins undergo posttranslational modifications catalyzed by three essential enzymes (Lgt, LspA, and Lnt). The mature lipoproteins are then sorted to the inner or outer membrane via the Lol system (LolABCDE). Recent studies suggested that this paradigm may not be universally applicable among different classes of proteobacteria. In this study, we conducted a systematic analysis of lipoprotein processing and sorting in Helicobacter pylori, a member of the *Epsilonproteobacteria* that colonizes the human stomach. We show that H. pylori *lgt*, *lspA*, and *lnt* homologs can complement conditionally lethal E. coli mutant strains in which expression of these genes is conditionally regulated. Mutagenesis studies and analyses of conditionally lethal H. pylori mutant strains indicate that *lgt* and *lspA* are essential for H. pylori growth but *lnt* is dispensable. H. pylori *lolA* and the single *lolC* (or *lolE*) homolog are also essential genes. We then explored the role of lipoproteins in H. pylori Cag type IV secretion system (Cag T4SS) activity. Comparative analysis of the putative VirB7 homolog CagT in wild-type and *lnt* mutant H. pylori strains indicates that CagT undergoes amino-terminal modifications consistent with lipidation, and we show that CagT lipidation is essential for CagT stability and Cag T4SS function. This work demonstrates that lipoprotein synthesis and localization in H. pylori diverge from the canonical pathways and that lipidation of a T4SS component is necessary for H. pylori Cag T4SS activity.

## Abstract (precis)

Bacterial lipoproteins have diverse roles in multiple aspects of bacterial physiology, antimicrobial resistance, and pathogenesis. Dedicated pathways direct the posttranslational lipidation and localization of lipoproteins, but there is considerable variation in these pathways among the proteobacteria. In this study, we characterized the proteins responsible for lipoprotein synthesis and localization in Helicobacter pylori, a member of the *Epsilonproteobacteria* that contributes to stomach cancer pathogenesis. We also provide evidence suggesting that lipidation of CagT, a component of the H. pylori Cag T4SS, is required for delivery of the H. pylori CagA oncoprotein into human gastric cells. Overall, these results constitute the first systematic analysis of H. pylori lipoprotein production and localization pathways and reveal how these processes in H. pylori differ from corresponding pathways in model proteobacteria.

## INTRODUCTION

Bacterial lipoproteins undergo posttranslational addition of acyl chains to their amino-terminal ends, which helps localize and anchor the mature lipoproteins (1). Lipoproteins have roles in multiple processes, including nutrient uptake, signal transduction, adhesion, conjugation, sporulation, antibiotic resistance, protein transport, and extracytoplasmic folding of proteins (2–7). In addition, bacterial lipoproteins present a pathogen-associated molecular pattern (PAMP) recognized by Toll-like receptor 2 (TLR2) when heterodimerized with either TLR1 or TLR6 (8). Recognition of lipoproteins through TLR2 stimulates the production of proinflammatory cytokines and antimicrobial effector molecules.

Lipoprotein synthesis in Gram-negative bacteria requires multiple steps (see Fig. S1 in the supplemental material) (9, 10). Newly synthesized lipoproteins are typically recognized and exported via the Sec pathway. The lipidation machinery then recognizes a short cysteine-containing amino acid sequence known as a lipobox, located near the amino terminus of the protein. The first modification is the addition of a diacylglyceride to the cysteine sulfhydryl of the preprolipoprotein, catalyzed by prolipoprotein diacylglyceryl transferase (Lgt). Amino acids preceding the cysteine are then cleaved by prolipoprotein signal peptidase (LspA, signal peptidase II), resulting in a diacylated apolipoprotein. Finally, the amino-terminal cysteine is N-acylated by apolipoprotein N-acyltransferase (Lnt) to produce the mature triacylated lipoprotein. The three lipoprotein-specific enzymes (Lgt, LspA, and Lnt) are essential for growth of the model organism, Escherichia coli. However, Wolbachia pipientis (a *Wolbachia* endosymbiont of Brugia malayi) lacks a *lnt* homolog, and mutational analyses demonstrated that *lnt* is not essential in Sinorhizobium meliloti (11–13) or Francisella tularensis or Neisseria gonorrhoeae (14) or in Acinetobacter species (15).

10.1128/mBio.00911-20.1 FIG S1 Posttranslational modifications in synthesis of lipoproteins in Gram-negative bacteria. The first modification is the addition of a diacylglyceride to the cysteine sulfhydryl of the preprolipoprotein, catalyzed by prolipoprotein diacylglyceryl transferase (Lgt). Amino acids preceding the cysteine are cleaved by prolipoprotein signal peptidase (Lsp), resulting in a diacylated apolipoprotein. Finally, a fatty acid is ligated to the amino terminus of the amino-terminal cysteine by apolipoprotein N-acyltransferase (Lnt) to produce the mature triacylated lipoprotein. Download FIG S1, PDF file, 0.1 MB. Copyright © 2020 McClain et al. 2020 McClain et al. This content is distributed under the terms of the Creative Commons Attribution 4.0 International license.

In the canonical lipoprotein sorting pathway in Gram-negative bacteria, newly synthesized lipoproteins destined for the outer membrane interact with the LolCDE complex in the inner membrane. Lipoproteins retained in the inner membrane include a “Lol avoidance signal” and do not interact with LolCDE (9). LolCDE transfers the newly synthesized lipoprotein to the periplasmic protein LolA, which then transfers the lipoprotein to the outer membrane lipoprotein LolB for insertion into the outer membrane. Additional proteins may then act to transfer a subset of outer membrane lipoproteins to the external leaflet of the outer membrane (16).

Recent studies indicate that lipoprotein sorting in some species of bacteria does not conform to the canonical Lol pathway (17, 18). For example, the *Alpha*- and *Betaproteobacteria*, as well as some *Gamma*-, *Delta*-, and *Epsilonproteobacteria*, harbor a single *lolF* gene encoding a protein that has features of both LolC and LolE (14). Though *lnt* is typically essential in bacteria containing LolC and LolE, *lnt* appears to be nonessential in bacteria containing LolF. In the absence of *lnt*, lipoproteins are expected to be diacylated. Thus, it has been hypothesized that LolF, in contrast to LolC and LolE, can recognize both diacylated and triacylated lipoproteins for sorting to the outer membrane (14, 15). As additional evidence that the canonical pathway may not be broadly applicable, *lolB* is not found in the *Alpha*- or *Epsilonproteobacteria* (16, 19). Furthermore, although *lolA* and *lolB* are essential in wild-type (WT) E. coli, either gene may be mutated in strains deficient in Lpp or OsmB, and global transposon mutagenesis of Caulobacter crescentus suggests that *lolA* is not essential (20, 21). Together, these results suggest that some bacterial classes utilize a lipoprotein sorting pathway distinct from the LolAB pathway (20).

Helicobacter pylori is a Gram-negative bacterium, classified among the *Epsilonproteobacteria*, that colonizes the gastric mucosa of humans (22–25). Colonization with H. pylori induces gastric mucosal inflammation and is associated with an increased risk for peptic ulcer disease, gastric adenocarcinoma, and gastric lymphoma (26–29). The H. pylori *cag* pathogenicity island (*cag* PAI) encodes a secreted effector protein (CagA) and a type IV secretion system (Cag T4SS) that delivers CagA into human gastric cells (30, 31). Individuals colonized with H. pylori strains harboring the *cag* PAI have a higher risk of gastric cancer or peptic ulcer disease than individuals colonized with *cag* PAI-negative strains or H. pylori-negative individuals (32). The H. pylori genome is predicted to encode approximately 20 lipoproteins, but these predictions are largely based on informatics-driven identification of putative lipoboxes (short peptide motifs containing the cysteine that becomes lipidated) (33–35). Experimental analysis of putative H. pylori lipoproteins has been hindered by an inability to label lipoproteins in H. pylori using ^3^H-palmitate, perhaps because H. pylori lacks proteins involved in long-chain fatty acid transport and catabolism (36–38), and by the failure of globomycin to inhibit signal peptide cleavage by H. pylori LspA (36, 39). Thus, characterization of H. pylori lipoproteins by recombinant expression in E. coli, coupled with ^3^H-palmitate incorporation or globomycin-mediated inhibition of LspA or both, has been undertaken for only a small number of H. pylori lipoproteins (36, 37, 39, 40). H. pylori lipoproteins are currently being investigated as antigens for potential inclusion in H. pylori vaccines (41–47) and are believed to have important functions in bacterial adhesion to mammalian cells and colonization of the stomach (37, 39, 48), altering cell migration and signaling (36, 49) and stimulating gamma interferon (IFN-γ) production (50) and natural transformation competence (51).

H. pylori homologs of the enzymes involved in posttranslational modification of lipoproteins (Lgt, Lsp, and Lnt) have been proposed, as have homologs of LolA and LolF (33, 52–54). However, it remains unclear whether these enzymes are essential for H. pylori growth. One study reported that *lspA* is essential whereas *lolA* is nonessential (54). Conversely, a microarray-based analysis of a transposon mutant library in H. pylori detected transposon insertions in *lgt*, *lspA*, *lnt*, and *lolF* but no insertions in *lolA* (55). A systematic experimental analysis of lipoprotein synthesis and localization pathways in H. pylori has not yet been performed.

In the present report, we present a comprehensive analysis of H. pylori lipoprotein synthesis and localization pathways. We report that the genes whose products are predicted to mediate posttranslational modification of H. pylori lipoproteins are able to complement E. coli mutants in which the corresponding E. coli genes were conditionally regulated. We constructed gene knockouts or conditional mutants in H. pylori and show that *lgt*, *lspA*, *lolA*, and *lolF* are essential for H. pylori growth whereas *lnt* is dispensable. We also provide experimental evidence that CagT (a component of the H. pylori Cag T4SS and a putative VirB7 homolog) undergoes amino-terminal modifications consistent with lipidation. By analyzing a *lnt* mutant, we show that there is little if any alteration of Cag T4SS activity when H. pylori lipoproteins (including CagT) were diacylated rather than triacylated, and by analyzing mutants in which the CagT lipobox was disrupted, we show that lipidation of CagT is essential for CagT stability and Cag T4SS activity.

## RESULTS

### Lipoprotein synthesis: functional complementation of conditionally lethal E. coli mutant strains.

**FIG 1:**
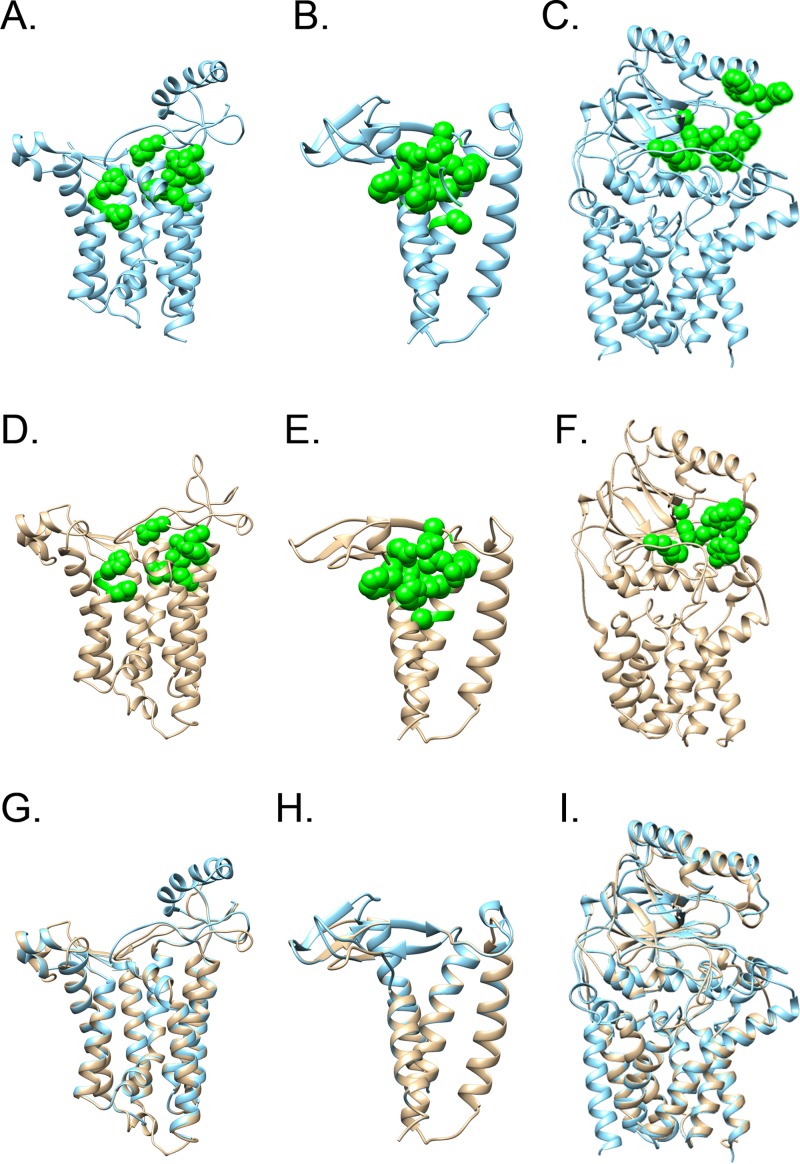
Conservation among lipoprotein synthetic enzymes Lgt, LspA, and Lnt. Ribbon diagrams representing E. coli Lgt (A) (PDB 5AZC [56]), P. aeruginosa LspA (B) (PDB 5DIR [59]), and E. coli Lnt (C) (PDB 5N6H [61]) and predicted structures of H. pylori Lgt (D), Lsp (E), and Lnt (F) (generated by submitting the H. pylori sequences to Phyre2 [92]) are shown. Superimposed structures of Lgt (G), LspA (H), and Lnt (I) were generated using Chimera (93). The amino acid side chains of important conserved residues are shown as green spheres.

H. pylori genomes are predicted to contain homologs of the canonical genes *lgt*, *lspA*, and *lnt* (genes *hp0955*, *hp0074*, and *hp0180*, respectively, in sequenced strain 26695) required for the posttranslational modification of lipoproteins in E. coli (Fig. 1). The predicted H. pylori proteins share a relatively low level of amino acid sequence identity with characterized homologs found in E. coli or Pseudomonas aeruginosa (about 20 to 40% amino acid identity). However, each of the H. pylori proteins is predicted to adopt a three-dimensional fold similar to that of respective counterparts in other bacteria, and critical residues in each enzyme are conserved (Fig. 1). Conserved residues in H. pylori 26695 Lgt include R155 and E163 (corresponding to E. coli Lgt R143 and E151), predicted to bind phosphatidyl glycerol, the HGGL motif (residues 115 to 118 of H. pylori Lgt) which may bind to the peptide substrate, and an H-bond network consisting of R155, R232, E236, and R239 (R143, R239, E243, and R246 in E. coli Lgt) predicted to catalyze the transfer of diacylglycerol to the preprolipoprotein (56–58). H. pylori 26695 LspA includes the catalytic dyad D114 and D131 (D124 and D143 in Pseudomonas aeruginosa LspA) and 10 of 12 additional residues strictly conserved in LspA proteins from 485 organisms (the remainder of the 12 residues, G108 and A109 in P. aeruginosa LspA, are replaced by A98 and G99 in H. pylori LspA) (59). Finally, conserved residues in H. pylori 26695 Lnt include a catalytic triad comprised of E242, K296, and C349 (E267, K335, and C387 in E. coli Lnt) as well as residues Q207, K210, F211, N371, and W374 (Q233, K236, W237, N412, and W415 in E. coli Lnt) that form a pocket surrounding the site where the phosphate head group of the phospholipid donor is predicted to bind (60–62).

**FIG 2:**
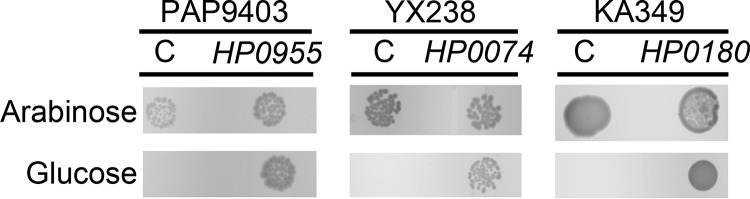
Functional properties of H. pylori *lgt*, *lsp*, and *lnt* expressed in E. coli. E. coli PAP9403, YX238, and KA349 contain *lgt*, *lspA*, and *lnt*, respectively, under the control of an arabinose-inducible promoter (58, 63, 64). Since expression of *lgt*, *lsp*, and *lnt* is essential for bacterial growth, these strains do not grow on medium containing glucose but do grow on medium containing arabinose. Introduction of plasmids containing the homologous H. pylori genes under the control of the *lacUV5* or Trc promoter supported growth on glucose. Vector-only control plasmids (C) did not support growth on glucose. Results are representative of 3 experiments.

To experimentally evaluate whether these H. pylori proteins function as predicted, we sought to complement E. coli mutant strains in which the expression of genes required for posttranslational modification of lipoproteins is conditionally regulated. In these mutant strains, the expression of *lgt*, *lspA*, or *lnt* is under the control of an arabinose-inducible promoter (58, 63, 64). These strains grew on media supplemented with arabinose but did not grow on media supplemented with glucose (Fig. 2), indicating that the regulated enzymes are essential for growth. We introduced plasmids encoding the predicted H. pylori homologs under the control of the *lacUV5* or *trc* promoter into the E. coli strains in which expression of *lgt*, *lspA*, or *lnt* is controlled by the arabinose-inducible promoter. In contrast to the parental strains, the strains containing these plasmids grew on media supplemented with glucose (Fig. 2). These results indicate that H. pylori *lgt*, *lspA*, and *lnt* homologs can functionally complement conditionally lethal E. coli *lgt*, *lspA*, and *lnt* mutant strains, respectively.

### Lipoprotein synthesis: construction and analysis of H. pylori mutant strains.

We next sought to determine whether the *lgt*, *lspA*, or *lnt* genes are essential in H. pylori. H. pylori *s*train 26695 was transformed with DNA constructs designed to disrupt *lgt*, *lspA*, or *lnt* by insertion of a gene conferring antibiotic resistance. Derivatives in which *lnt* was disrupted were readily isolated (e.g., VM211), indicating that *lnt* is not essential for H. pylori growth. In contrast, we were unable to isolate strains in which *lgt* or *lspA* was disrupted, despite repeated attempts.

**FIG 3:**
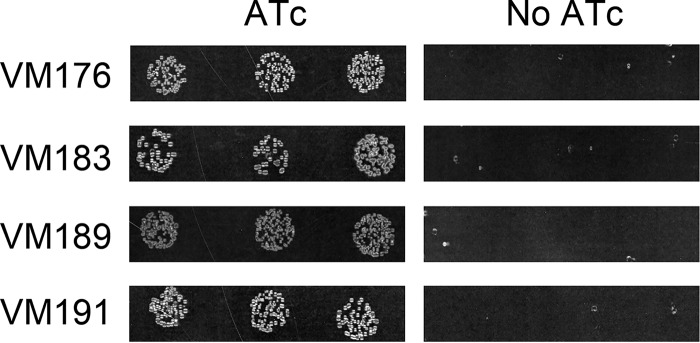
Growth of H. pylori conditional mutants. H. pylori strains engineered to express *lgt* (VM176), *lspA* (VM183), *lolA* (VM189), or *lolF* (VM191) under the control of a TetR-regulated promoter were inoculated onto media in the presence or absence of anhydrotetracycline (ATc). Representative results from three independent cultures of each mutant are shown.

As a complementary approach for assessing the essentiality of these genes, we constructed derivatives of H. pylori strain 26695 in which *lgt* or *lspA* was placed under the control of a TetR-regulated promoter (65). We then compared the growth of the resulting strains, VM176 and VM183, in the absence or presence of anhydrotetracycline (ATc), which results in repressed or derepressed expression of the gene of interest, respectively. Results indicated that strains containing TetR-regulated copies of *lgt* or *lspA* grew in the presence but not in the absence of ATc, indicating that *lgt* and *lspA* are essential for H. pylori growth (Fig. 3).

**FIG 4:**
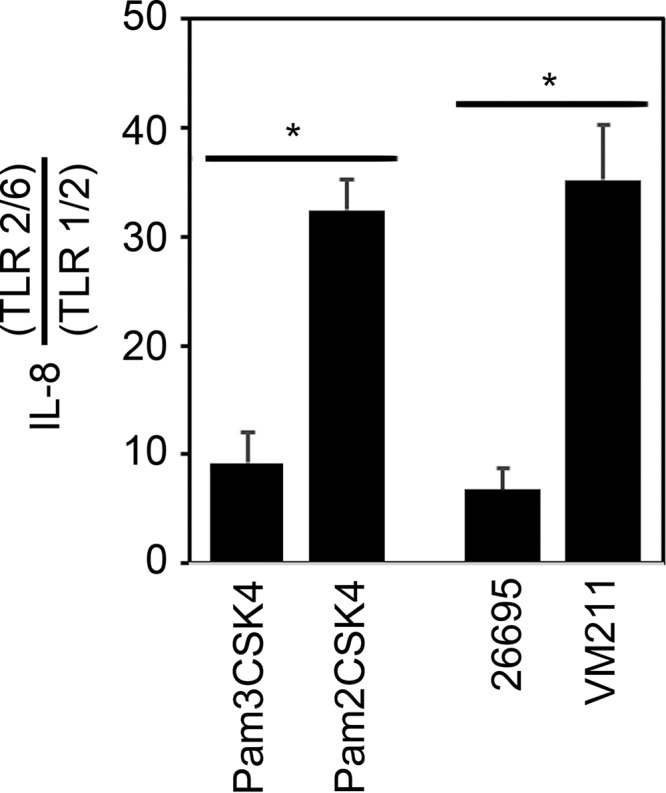
TLR2 activation by H. pylori lipoproteins. Protein extracts enriched in lipoproteins were prepared from H. pylori strain 26695 or the *lnt* mutant strain VM211 and were added (150 ng per ml) to 293-mTLR1/2 or 293-hTLR2/6 cell lines. As controls, cells also were incubated with triacylated Pam3CSK4 or diacylated Pam2CSK4 (30 ng per ml). Following 24 h of incubation, culture supernatants were recovered and subjected to ELISA to determine IL-8 concentrations. Results are expressed as the ratio of the level of IL-8 produced by 293-hTLR2/6 cells divided by the level of IL-8 produced by 293-hTLR1/2 cells and represent means and standard deviations of three independent experiments, each with triplicate samples. Asterisks denote results that were significantly different from the control results (Student's *t* test, *P* < 0.0003).

Lipoproteins represent pathogen-associated molecular patterns that can be recognized by TLR2 heterodimers on host cells. Triacylated lipoproteins are recognized by TLR1/TLR2 heterodimers, whereas diacylated lipoproteins are recognized by TLR2/TLR6 heterodimers. Upon binding of lipoproteins to TLR2 heterodimers (8), host cells respond by activation of NFκB-responsive promoters, including promoters for genes encoding proinflammatory cytokines such as interleukin-8 (IL-8). To compare the TLR2-activating properties of wild-type (WT) and *lnt* mutant H. pylori, we used HEK293 cell lines stably transfected to express human TLR1/TLR2 or mouse TLR2/TLR6 heterodimers. Addition of lipoprotein-enriched protein extracts from WT H. pylori to TLR2-expressing cells elicited responses similar to the responses induced by the triacylated control peptide Pam3CSK4 (Fig. 4). In contrast, addition of lipoprotein-enriched protein extracts prepared from *lnt* mutant VM211 elicited responses similar to the responses induced by the diacylated control peptide Pam2CSK4 (Fig. 4). These results are consistent with the presence of triacylated lipoproteins in WT H. pylori and the presence of diacylated lipoproteins in *lnt-*deficient H. pylori.

### Lipoprotein localization genes: construction of H. pylori mutant strains.

Of the five genes whose products direct lipoprotein localization in E. coli (*lolABCDE*), H. pylori appears to lack *lolB* (16, 19), and a homolog of *lolD* has not been identified. One of numerous predicted ABC transporter-like ATP-binding proteins likely encodes LolD (53). H. pylori is predicted to encode a homolog of *lolA* (*HP0785*) as well as a single homolog of *lolC* or *lolE* (which has been termed *lolF* [*HP0787*]) to direct localization of newly synthesized lipoproteins (14). We sought to determine whether the *lolA or lolF* genes are essential in H. pylori. Repeated attempts to isolate strains in which either *lolA* or *lolF* was disrupted by insertion of a gene conferring antibiotic resistance were unsuccessful. As an alternative approach for assessing the essentiality of these genes, we constructed derivatives of strain 26695 in which *lolA* or *lolF* was placed under the control of a TetR-regulated promoter (65). The resulting strains, VM189 and VM191, were able to grow in the presence of ATc, but growth was arrested when the bacteria were cultured on media lacking ATc (Fig. 3). These results indicate that *lolA* and *lolF* are essential in H. pylori.

### H. pylori Cag T4SS activity and posttranslational lipidation.

**FIG 5:**
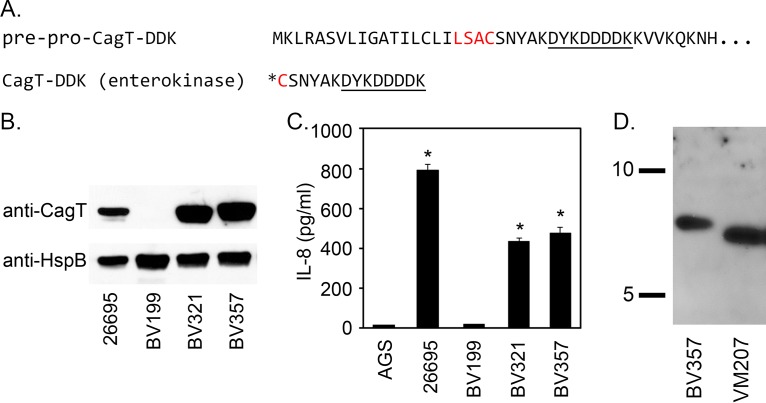
Analyses of CagT-DDK. (A) Amino-terminal amino acid sequence of CagT-DDK and the CagT-DDK peptides following enterokinase treatment. The lipobox is highlighted in red, the DDK epitope is underlined, and the asterisk indicates the site of triacyl lipid modification in WT H. pylori or the site of diacyl lipid modification in *lnt* mutant H. pylori. Signal peptide cleavage occurs between the “A” and “C” within the lipobox. Enterokinase cleavage occurs after lysine at its cleavage site DDDDK. (B) Expression of CagT was assessed by immunoblotting extracts of strains 26695 (WT), BV199 (Δ*cagT*), BV321 (restored *cagT*), and BV357 (*cagT-DDK_27_*) using anti-CagT and anti-HspB (as loading control). (C) H. pylori strains were cocultured with AGS cells, and the ability of each strain to induce IL-8 production was determined by ELISA. Asterisks denote results that were significantly different from the BV199 control results (analysis of variance [ANOVA] followed by Dunnett’s *post hoc* test, *P* < 0.05). (D) Protein extracts from BV357 and VM207 (each producing CagT-DDK_27_, the latter in an *lnt* mutant background) were treated with enterokinase and immunoblotted using anti-DDK monoclonal antibody. Consistent with expectations, the immunoreactive peptides from BV357 and VM207 differed in molecular mass.

Secretion of the oncoprotein CagA and other effector molecules by H. pylori is mediated through a type IV secretion system (Cag T4SS) (66). Several of the H. pylori proteins required for T4SS activity exhibit sequence relatedness to components of T4SSs in other bacterial species. These include CagT, a putative homolog of the lipoprotein VirB7 (67). CagT (32 kDa) is much larger in size than VirB7 proteins from most bacterial species (for example, Agrobacterium tumefaciens VirB7 is 6 kDa), and comparisons of CagT with putative VirB7 homologs showed a very low level of amino acid sequence relatedness (31). To evaluate whether CagT undergoes N-terminal modifications consistent with lipidation, we introduced a gene encoding CagT with a DDK epitope between amino acids 26 and 27 into an H. pylori strain harboring a deletion of the endogenous *cagT* gene (Fig. 5A). The resulting strain, BV357, expressed CagT-DDK and retained Cag T4SS function (Fig. 5B and C). We next deleted *lnt* from the CagT-DDK strain, resulting in strain VM207. Protein extracts were prepared from BV357 and VM207, and the extracts were treated with enterokinase (which cleaves at the C-terminal end of the DDK epitope) (Fig. 5A). Immunoblotting the resulting protein preparations with anti-DDK antibody revealed the presence of DDK-reactive peptides in both BV357 and VM207, each with an apparent molecular mass somewhat higher than expected (predicted molecular weights for the triacylated and diacylated peptides are 2.5 and 2.2 kDa, respectively). Anomalous SDS-PAGE migration of lipoproteins is a common phenomenon (68–71). The DDK-reactive peptide from *lnt* mutant strain VM207 has a lower molecular mass than the peptide from strain BV357, consistent with a failure to add the third acyl chain in the absence of *lnt* (Fig. 5). These results are consistent with the presence of an Lnt-dependent N-terminal posttranslational modification of CagT.

**FIG 6:**
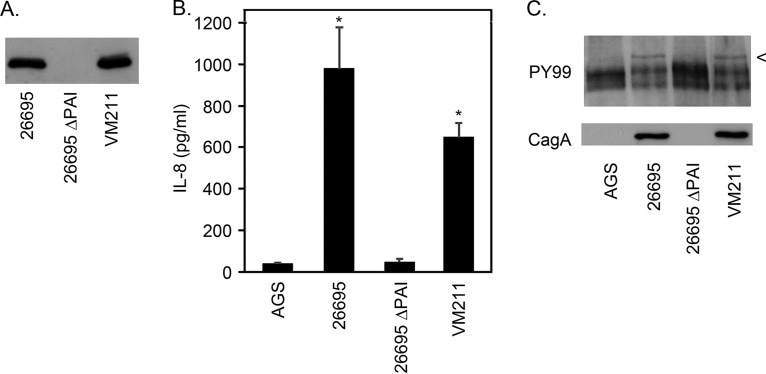
Activity of diacylated CagT. (A) Whole-cell lysates from H. pylori strains 26695, 26695 ΔPAI, and VM211 (Δ*lnt*) were analyzed by immunoblotting with anti-CagT antisera. A representative blot is shown. (B and C) AGS cells were cultured alone or in the presence of H. pylori strains at an MOI of 100:1 for 7 h. Cell culture supernatants were analyzed for IL-8 by ELISA (B), and cell lysates were analyzed by immunoblotting (C) using an antibody recognizing phospho-Tyr (PY99) to detect phosphorylated CagA (indicated by an arrowhead) or antiserum directed against CagA (to detect total CagA). Multiple additional bands (unrelated to CagA) were detected by the anti-phospho-Tyr antibody in all samples, including AGS cells alone. Results in panel B represent means and standard deviations of three biological replicates, each analyzed in triplicate; results in panel C are representative of three biological replicates. Asterisks denote results that were significantly different from the 26695 ΔPAI control results (ANOVA followed by Dunnett’s *post hoc* test, *P* < 0.05).

We next analyzed Cag T4SS function in wild-type H. pylori 26695 and the *lnt* mutant strain (VM211). Immunoblotting demonstrated that the steady-state levels of CagT were similar in 26695 and VM211 (Fig. 6A). Cocultured with AGS cells, both 26695 and VM211 were able to induce IL-8 production (a phenotype dependent on Cag T4SS activity), whereas a control strain lacking the *cag* PAI was not (Fig. 6B). Furthermore, both 26695 and VM211 were able to translocate the effector protein CagA into AGS cells, where the protein then became phosphorylated (Fig. 6C). These results suggest that there is little if any alteration of Cag T4SS activity when H. pylori lipoproteins (including CagT) are diacylated rather than triacylated.

**FIG 7:**
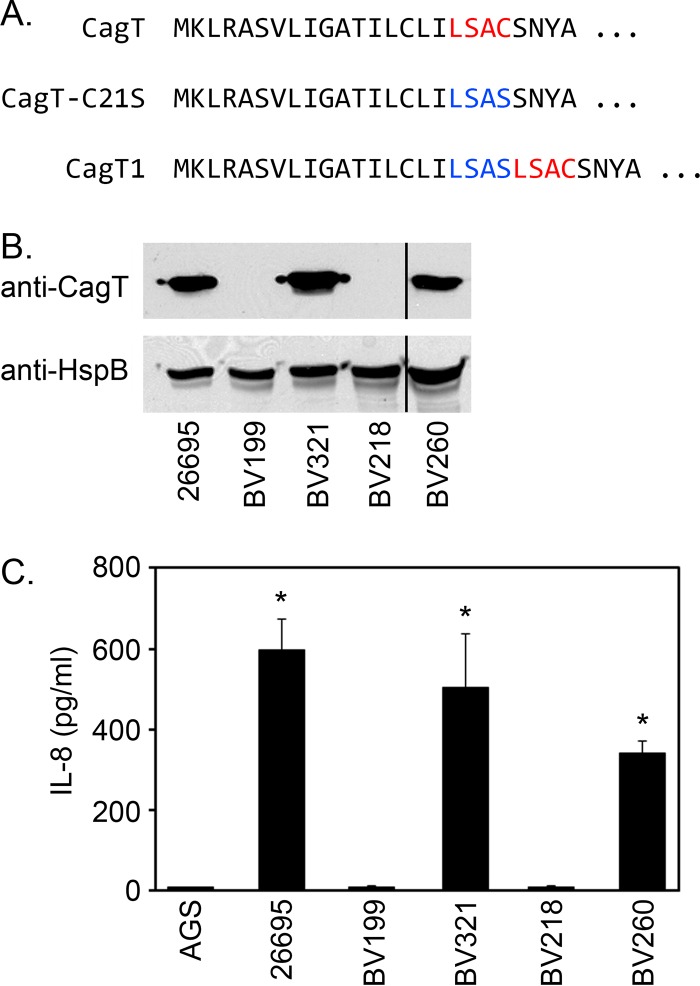
Requirement of a lipobox cysteine residue for CagT stability. (A) Amino-terminal amino acid sequences of CagT and mutant forms of CagT analyzed in this study. The CagT lipobox is highlighted in red, and the disrupted lipobox (CagT-C21S) is highlighted in blue. (B) Expression of CagT was evaluated by immunoblotting extracts of strains 26695 (WT), BV199 (Δ*cagT*), BV321 (in which a WT copy of *cagT* was introduced into the *ureA* locus-restored *cagT* strain), BV218 (*cagT-C21S*), and BV260 (*cagT1*) using anti-CagT. Vertical line indicates cropping of an additional unreported lane from the image. (C) H. pylori strains were cocultured with AGS cells, and the ability of each strain to induce IL-8 production was determined by ELISA. Asterisks denote results that were significantly different from the BV199 control results (ANOVA followed by Dunnett’s *post hoc* test, *P* < 0.05).

To determine whether lipidation of CagT is required for Cag T4SS function, we constructed H. pylori strains expressing mutant forms of *cagT* in which the putative CagT lipobox (a peptide motif within the signal peptide containing an essential cysteine as the site of lipidation) was disrupted (by introducing a C21S mutation, strain BV218) or restored (strain BV260) (Fig. 7). Analyses of steady-state levels of CagT by immunoblotting revealed that disruption of the putative lipobox led to destabilization of CagT whereas introduction of an intact lipobox after the C21S mutation (CagT1) resulted in wild-type levels of CagT (Fig. 7). Consistent with these findings, the C21S mutation abolished the ability of H. pylori to induce IL-8 expression in AGS gastric epithelial cells (Fig. 7).

**FIG 8:**
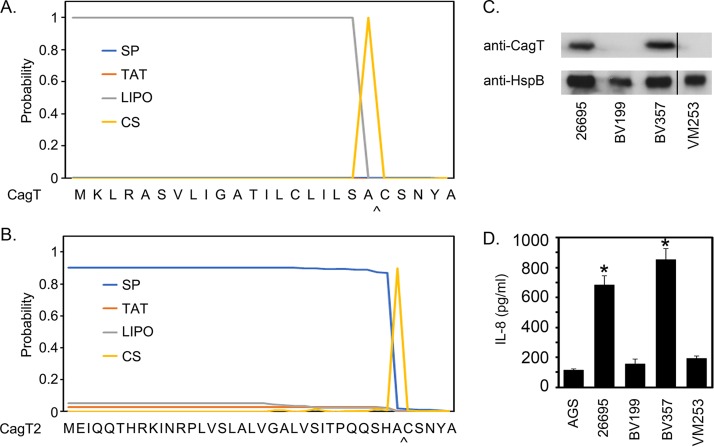
An intact lipobox is required for CagT stability. (A and B) SignalP 5.0 predicts signal peptide cleavage of wild-type CagT and a mutant CagT protein (CagT2) in which the H. pylori VacA signal peptide was fused to CagT (72). The sites of predicted signal peptide cleavage by LspA (LIPO) or by signal peptidase I (SP) and the corresponding cleavage sites (CS) are shown. (C) Immunoblot showing steady-state levels of CagT 26695 (WT), BV199 (Δ*cagT*), BV357 (CagT-DDK), and VM253 (*cagT2*) determined using anti-CagT. Vertical line indicates cropping of an additional unreported lane from the image. (D) H. pylori strains were cocultured with AGS cells, and the ability of each strain to induce IL-8 production was determined by ELISA. Asterisks denote results that were significantly different from the BV199 control results (ANOVA followed by Dunnett’s *post hoc* test, *P* < 0.05).

One possible explanation for the results shown in Fig. 7 is that the CagT-C21S mutant protein may lack a functional signal peptide and therefore may not be properly secreted. Analysis of CagT by SignalP-5.0 predicts signal peptide cleavage by LspA between Ala20 and Cys21 (Fig. 8A) (72). In contrast, analysis of CagT-C21S predicts no signal peptide cleavage (data not shown). Therefore, as a complementary approach, we constructed an H. pylori strain, VM253, expressing a mutant protein (CagT2) in which the 20-amino-acid signal peptide of CagT was replaced with the 33-amino-acid signal peptide from the secreted protein VacA (Fig. 8A and B). This mutant CagT also includes a DDK epitope between amino acids 26 and 27. Both wild-type CagT and the CagT2 chimera are expected to undergo signal peptide cleavage immediately preceding a Cys residue (C21 in WT CagT, C34 in CagT2) (Fig. 8A and B). However, the mutant CagT2 is not expected to undergo lipidation due to the absence of a lipobox (Fig. 8B). Immunoblotting of protein extracts from strain VM253 revealed that exchange of the native CagT signal peptide with the signal peptide from VacA led to destabilization of CagT (Fig. 8C). Correspondingly, strain VM253, expressing the mutant protein (CagT2), was defective in inducing IL-8 expression in AGS gastric epithelial cells (Fig. 8D). Therefore, the combined results of Fig. 7 and Fig. 8 indicate that an intact CagT lipobox is required for CagT stability and Cag T4SS activity.

## DISCUSSION

Genes *HP0955*, *HP0074*, and *HP0180* in H. pylori strain 26695 are annotated as homologs of *lgt*, *lspA*, and *lnt*, respectively (33, 52–54). Although there is very limited primary amino acid sequence similarity between the encoded H. pylori proteins and more thoroughly characterized homologs from other species, multiple amino acids predicted to be required for functional activity are conserved (Fig. 1). In the present study, we report that H. pylori *HP0955*, *HP0074*, and *HP0180* are indeed functional homologs of *lgt*, *lspA*, and *lnt*, based on the ability of each gene to complement conditionally lethal E. coli mutant strains.

Results of previous studies have provided conflicting evidence on whether genes involved in lipoprotein synthesis and sorting in H. pylori are essential (54, 55). The comprehensive analyses conducted in the present study indicated that the *lgt* and *lspA* genes encoding lipoprotein synthetic enzymes as well as the genes whose products direct lipoprotein localization, *lolA* and *lolF*, are essential in H. pylori. In contrast, *lnt* is not essential. The latter result is consistent with recent studies suggesting that *lnt* is not essential in bacterial species containing *lolF* in place of *lolC* and *lolE* (14, 15). The affinity of the LolCDE complex is much higher toward triacylated lipoproteins than diacylated lipoproteins, and this likely explains the essential nature of *lnt* in species containing *lolC* and *lolE* (73). In contrast, the nonessential nature of *lnt* in species containing *lolF* suggests that the affinity of LolF toward diacylated lipoproteins (the product of Lgt action) is sufficient to direct them to LolA for sorting.

In the canonical sorting pathway, lipoproteins destined for localization to the outer membrane are transferred from the inner membrane LolCDE complex to the periplasmic LolA protein. LolA then transfers the lipoproteins to LolB anchored in the outer membrane (9). Previous studies have concluded that LolA receives lipoproteins from LolCDE but cannot insert lipoproteins into membranes (74–78), and similar studies have concluded that LolB can insert lipoproteins into membranes but cannot accept lipoproteins from LolCDE (9, 78, 79). There are several possible explanations for the essentiality of *lolA* in H. pylori and the lack of a recognizable H. pylori *lolB* (and for the similar apparent absence of *lolB* in the *Alpha*- and other *Epsilonproteobacteria* [16, 19]), including the following: (i) outer membrane lipoproteins are not essential in H. pylori; (ii) H. pylori LolA, unlike E. coli LolA, can insert lipoproteins into membranes; (iii) there is an uncharacterized protein in H. pylori with a function analogous to that of LolB; and/or (iv) H. pylori possesses a LolAB-bypass system, as has recently been suggested in E. coli (20).

H. pylori strains lacking CagT (either through *cagT* knockout mutation or with repressed *cagT* gene expression) are deficient in Cag T4SS activity (65, 80, 81), and we now present experimental evidence indicating that CagT undergoes N-terminal modification consistent with lipidation. Lipidation of CagT is necessary for CagT stability and therefore for Cag T4SS activity. Comparisons of Cag T4SS activities in wild-type and mutant H. pylori in which lipidation of CagT is disrupted suggest that CagT functions similarly whether it is triacylated or diacylated. However, disruption of CagT lipidation appears to destabilize CagT, most likely due to defects in secretion or membrane anchoring leading to degradation of the mislocalized protein.

**FIG 9:**
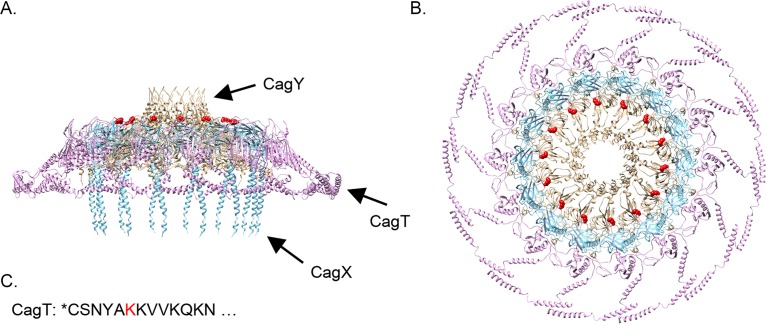
The lipidated amino-terminal end of CagT is positioned at the interface between the Cag T4SS and the H. pylori outer membrane. (A and B) Ribbon representations of CagT, CagX, and CagY (PDB accession no. 6OEE, 6OEG, and 6OEF, respectively [31]) within the Cag T4SS outer membrane core complex are shown in side (A) and top (B) views. The amino-terminal residue resolved in the CagT structure is lysine 26 and is highlighted in red. (C) The amino-terminal amino acid sequence of CagT is shown with an asterisk indicating the site of lipidation, and lysine 26 is highlighted in red.

Previous studies demonstrated that CagT is essential for Cag T4SS assembly and function (65, 80, 81). Analyses of the Cag T4SS structure by electron cryotomography (cryo-electron tomography [cryo-ET]) revealed that outer membrane core complexes from *cagT* mutant strains were highly variable in structure, generally consisting of a central ring but lacking the peripheral densities seen in intact complexes (82). A reduced number of core complexes were visualized by cryo-ET in a *cagT* mutant strain compared to a WT strain, and a reduced number of core complexes were purified from the *cagT* mutant strain (82, 83); this might be attributable to reduced stability or impaired localization of the complexes in a *cagT* mutant strain. Single-particle cryo-electron microscopy analysis of Cag T4SS outer membrane core complexes suggested that the lipidated amino-terminal end of CagT is positioned to interact with the H. pylori outer membrane (Fig. 9) (31). Together, these results suggest that lipidated CagT helps stabilize the core complex and direct it or anchor it to the outer membrane.

In summary, this report provides a systemic experimental analysis of lipoprotein synthesis and localization pathways in H. pylori, a member of the *Epsilonproteobacteria*. The lipoprotein synthetic enzymes Lgt and LspA are essential, implying that one or more lipoproteins are essential for H. pylori. Lnt (which performs the final enzymatic step in the synthesis of triacylated lipoproteins in Gram-negative bacteria) is nonessential in H. pylori. This is consistent with previous reports indicating that Lnt is not required by certain members of the *Alpha*-, *Beta*-, and *Gammaproteobacteria* (11–15). The nonessential nature of Lnt in H. pylori is consistent with current understanding that Lnt is not required by bacteria (such as H. pylori) that produce LolF rather than LolC and LolE (14, 15). Further differentiating lipoprotein sorting in H. pylori from model proteobacteria is the observation that H. pylori, like other members of the *Epsilonproteobacteria* as well as the *Alphaproteobacteria*, appears to lack LolB (16, 19). The current study showed that lipidation of CagT is essential for Cag T4SS function, but we did not detect any substantial difference in T4SS activity dependent on whether CagT was diacylated or triacylated. In future studies, it will be important to determine what fitness advantage is provided by the nonessential *lnt*, to determine how CagT and other lipoproteins are directed to the H. pylori outer membrane in the absence of a *lolB* homolog, and to define the structural basis by which a lipid moiety in CagT helps direct or anchor the T4SS outer membrane core complex to the outer membrane.

## MATERIALS AND METHODS

### Bacterial strains and culture conditions.

Strains and plasmids used in this study are listed in Table 1. H. pylori strain 26695 was grown on either Trypticase soy agar (TSA) plates containing 5% sheep blood or in bisulfite-free *Brucella* broth containing 10% fetal bovine serum (BB-FBS) at 37°C in room air supplemented with 5% CO_2_. H. pylori mutant strains were selected using metronidazole (15 μg ml^−1^), kanamycin (12.5 μg ml^−1^), or chloramphenicol (2.5 μg ml^−1^). Anhydrotetracycline (ATc) was added as indicated at 100 ng ml^−1^ (65). Plasmids were maintained using E. coli strain DH5α or strain XL-10 Gold. E. coli strains were grown on Luria-Bertani (LB) agar plates or in LB broth containing ampicillin (100 μg ml^−1^), chloramphenicol (25 μg ml^−1^), or kanamycin (25 μg ml^−1^).

**TABLE 1:** Plasmids and strains used in this study

Plasmid or strain	Description or genotype^*a*^	Reference or source
Plasmids		
pAD1	Vector for introducing DNA into the H. pylori *ureA* locus	94, 95
pBbA5a-RFP	p15A origin, *bla*, *lacI*, *rfp*, *lacUV5* promoter	96
pBbA5c-RFP	p15A origin, *cat*, *lacI*, *rfp*, *lacUV5* promoter	96
pBbE1c-RFP	colE1 origin, *cat*, *lacI*, *rfp*, *trc* promoter	96
pBV173	H. pylori genome region that includes *cagT* cloned into pGEMT-Easy (Promega)	Current study
pBV175	pBV173 Δ*cagT*	Current study
pBV193	pGEMT-*cagT*-C21S	Current study
pBV253	pGEMT-*cagT1*	Current study
pBV334	Derivative of pBV173 in which sequences encoding a DDK epitope are inserted into *cagT* after lysine 26	Current study
pBV342	H. pylori *cagT* cloned into pAD1	Current study
pBV449	H. pylori *lgt* cloned into pBbA5a-RFP	Current study
pMM690	H. pylori *lspA* cloned into pBbE1c-RFP	Current study
pMM691	H. pylori *lnt* cloned into pBbA5c-RFP	Current study
pMM693	*pAD1-cagT2*	Current study

Strains		
H. pylori 26695		33
H. pylori 26695 ΔPAI	26695 Δ(*HP520*–*547*)	89
H. pylori 26695 Δr*dxA*	26695 Δ*rdxA*	84
H. pylori 26695 Δr*dxA cagT*::*cat*/*rdxA*)	26695 Δ*rdxA cagT*::*cat*/*rdxA*	80
H. pylori BV199	26695 Δ*rdxA* Δ*cagT*	Current study
H. pylori BV218	26695 Δ*rdxA cagT-C21S*	Current study
H. pylori BV260	26695 Δ*rdxA cagT1*	Current study
H. pylori BV321	26695 Δ*rdxA* Δ*cagT ureA*::(*cagT* Chl^r^)	Current study
H. pylori BV357	BV199 *ureA*::*cagT*-DDK_26_	Current study
H. pylori VM124	26695 *ureA*::*tetR* Chl^r^	65
H. pylori VM165	26695 *ureA*::*tetR* Kan^r^	Current study
H. pylori VM173	VM165 *tetO*::*PcagU-lgt* (630–631, Chl^r^)	Current study
H. pylori VM176	VM173 Δ*lgt-rdxA* (Mtz^r^)	Current study
H. pylori VM183	VM124 *tetO-PcagU*::*lspA* Kan^r^	Current study
H. pylori VM189	VM124 *PcagU*::*lolA* Kan^r^	Current study
H. pylori VM191	VM124 *PcagU*::*lolF* Kan^r^	Current study
H. pylori VM207	BV357 Δ*lnt* Kan^r^	Current study
H. pylori VM211	26695 Δ*lnt* Kan^r^	Current study
H. pylori VM253	BV199 *ureA*::*cagT2* (Chl^r^)	Current study
E. coli DH5α	F^–^ Φ80*lacZ*ΔM15 Δ(*lacZYA-argF*) *U169 recA1 endA1 hsdR17* (r_K_^–^ m_K_^+^) *pho*A *sup*E44 λ^–^ *thi-1 gyrA96 relA1*	
E. coli PAP9403	*lacI*^q^ *rrnB*_T14_ Δ*lacZ*_WJ16_ *hsdR514* Δ*araBAD*_AH33_ Δ*rhaBAD*_LD78_ *lgt*::Kan^r^ pCHAP9231	58
E. coli YX238	MG1655 Δ*lspA*::Kan pBAD30::*lspA^Ec^*	63
E. coli KA349	*ybeX*-(Kan^r^-*rrnB* TT-*araC*-P_BAD_)-*lnt lpp*::Chl^r^	64
E. coli XL-10 Gold	*endA1 glnV44 recA1 thi-1 gyrA96 relA1 lac* Hte Δ(*mcrA*)*183* Δ(*mcrCB*-*hsdSMR*-*mrr*) *173* Tet^r^ F'[*proAB lacI*^q^ZΔM15 Tn*10*(Tet^r^ Amy Chl^r^)]	

a Chl^r^, chloramphenicol resistance; Kan^r^, kanamycin resistance; Mtz^r^, metronidazole resistance; Tet^r^, tetracycline resistance.

### Complementation of E. coli strains with H. pylori *lgt*, *lspA*, *and lnt*.

E. coli strains PAP9403, YX238, and KA349 are conditionally lethal strains in which *lgt*, *lspA*, and *lnt* (respectively) are expressed under the control of arabinose-inducible promoters (58, 63, 64). Expression of the relevant gene in these strains is stimulated by the presence of arabinose and inhibited by the addition of glucose. The *lgt*, *lspA*, *and lnt* genes from H. pylori 26695 were subjected to PCR amplification and cloned into pBbA5a-RFP (pBbA5a-red fluorescent protein), pBbE1c-RFP, and pBbA5c-RFP (Addgene), using EcoRI and BglII restriction sites, to yield pBV449, pMM690, and pMM691, respectively. These plasmids then were transformed into the relevant E. coli mutant strains. E. coli strains were cultured with isopropyl β-d-1-thiogalactopyranoside (IPTG) (Roche) (0.1 mM) and either arabinose (0.2%) or glucose (0.2%).

### Construction of recombinant H. pylori strains.

H. pylori strain 26695 was transformed with synthetic constructs (GenScript) designed to delete *lgt*, *lspA*, *lnt*, *lolA*, or *lolF*. For *lgt*, the construct included a deletion of *lgt* (HP0955) and *rdxA* (HP0954); deletion of *rdxA* confers metronidazole resistance (84). For *lspA*, *lnt*, *lolA*, and *lolF*, the genes of interest were replaced with an antibiotic resistance determinant. We succeeded only in obtaining H. pylori transformants in which *lnt* was deleted, resulting in strain VM211.

To place *lgt* under TetR regulation, *tetR* linked to a kanamycin resistance determinant was inserted into the *ureA* locus of strain 26695 to yield strain VM165. Then, a synthetic construct (GenScript) in which a chloramphenicol resistance determinant and 3 copies of *tetO* linked to the promoter of *cagUT* (65) placed upstream of HP0955 (and changing the ATG start codon to a TTG start codon [85]) was introduced into the intergenic region between HP0630 and HP0631 to yield strain HP173. Finally, the endogenous *lgt* locus was deleted using the *lgt* deletion construct described above to yield strain VM176.

To place *lspA*, *lolA*, or *lolF* under TetR regulation, strain VM124 (which contains TetR in the *ureA* locus) (65) was transformed with synthetic constructs in which a kanamycin resistance determinant and 3 copies of *tetO* linked to the promoter of *cagUT* (65) were placed upstream of *lspA*, *lolA*, and *lolF* to yield strains VM183, VM189, and VM191, respectively. The start codons for *lolA* (ATG) and *lspA* (GTG) were changed to TTG (85); *lolF* naturally begins with a TTG start codon.

To delete *cagT* from H. pylori, a DNA sequence containing *cagT* along with 500 bp of flanking sequences was amplified from H. pylori and ligated into pGEMT-Easy (Promega) to yield pBV173. Using inverse PCR, *cagT* was excised to produce pBV175. H. pylori 26695Δ*rdxA* (*cagT*::*cat*/*rdxA*) (80) was then transformed with pBV175, and metronidazole-resistant transformants were selected to yield H. pylori BV199, which contains an unmarked Δ*cagT* mutation. The *cagT* mutation in BV199 was complemented in *cis* by transformation with pBV342 (*ureA*::*cagT*-CAT) to produce strain BV321.

To generate an H. pylori strain expressing DDK-tagged CagT, pBV173 was mutagenized to insert DDK epitope-encoding sequences after the codon for lysine 26 of *cagT*, yielding pBV334. Plasmid pBV334 was then used to transform H. pylori strain 26695 Δ*rdxA cagT*::*cat*/*rdxA* (80), and metronidazole-resistant transformants were selected to yield H. pylori strain BV357. The *lgt* locus was deleted from strain BV357 using the *lgt* deletion construct described above to yield strain VM207.

To generate an H. pylori strain expressing the 33-amino-acid signal peptide from H. pylori VacA fused to CagT at Cys21 and including a DDK epitope inserted after amino acid 26 of CagT, a chimeric sequence including a chloramphenicol resistance determinant was synthesized by GenScript and cloned into plasmid pAD1. The resulting plasmid, pMM692, was used to transform H. pylori strain BV199, and chloramphenicol-resistant colonies were selected. The resulting strain was designated VM253.

Site-directed mutagenesis of *cagT* was accomplished using a QuikChange-Multi site-directed mutagenesis system (Agilent) and pBV173 as the template. The resulting plasmids (pBV193 [encoding CagT-C21S] and pBV253 [encoding CagT1]) were then used to transform H. pylori 26695 Δ*rdxA cagT*::*cat*/*rdxA* (80), and metronidazole-resistant transformants were selected. The resulting H. pylori strains were designated BV218 and BV260, respectively (Table 1).

### Analysis of CagT lipidation in H. pylori.

To evaluate N-terminal modifications of CagT in H. pylori, protein extracts were prepared from strains BV357 and VM207 (described above). H. pylori strains were cultured on TSA plates containing 5% sheep blood for 24 h. Bacteria were resuspended in phosphate-buffered saline (PBS), pelleted, and washed in PBS. Bacterial pellets were resuspended in PBS containing 0.5% SDS. Insoluble material was pelleted at 21,000 × *g* for 5 min. Proteins in the soluble fraction were recovered using methanol-chloroform and solubilized in 0.1% SDS (86). Protein concentrations were determined by microBCA assay (Pierce), and 50 μg of each extract was incubated with 2 units of enterokinase (EK Max; ThermoFisher) at 37°C for 4 h. Samples then were separated by Tricine–SDS-PAGE using a 16%T/3%C gel containing 6 M urea (87) and transferred to polyvinylidene difluoride (PVDF). The PVDF membrane was developed using mouse monoclonal anti-DDK antibody (clone M2; Sigma) followed by anti-mouse horseradish peroxidase (HRP) (Promega) and chemiluminescence detection (Pierce).

### TLR2 activation assay.

Protein extracts enriched in lipoproteins were prepared using Triton X-114 (TX114) (88). H. pylori strains were cultured in broth for 20 h. Cultures were pelleted and washed in PBS, and the bacterial pellets were resuspended in PBS containing 2% TX114 and incubated at 4°C with continuous mixing for 24 h. Insoluble material was pelleted, and the protein extract was warmed and pelleted at 37°C to promote separation of TX114 and aqueous phases. The upper, aqueous phase was discarded, and the lower, detergent phase was extracted two times with the starting volume of PBS. Proteins in the final detergent phase were precipitated with 4× volumes of −20°C acetone. The protein pellets were resuspended in PBS, and the protein concentrations were determined by micro-bicinchoninic acid (BCA) assay (Pierce).

293-mTLR1/2 (m, mouse) and 293-hTLR2/6 (h, human) cells (InvivoGen) were grown in DMEM with glucose (4.5 g liter^−1^), 2 mM l-glutamine, penicillin (50 U ml^−1^), streptomycin (50 μg ml^−1^), 10% FBS, and 10 μg ml^−1^ at 37°C with 5% CO_2_. These cells are stably transfected to express mouse TLR1 and TLR2 and to express human TLR2 and TLR6, respectively. Cells were seeded into 96-well tissue culture dishes at 2.5 × 10^4^ cells per well. Serial dilutions of either bacterial TX114 lipoprotein extracts or synthetic control peptides (triacylated lipoprotein Pam3CSK4 or diacylated Pam2CSK4; InvivoGen) were added to each well. Culture supernatant was collected after cells were stimulated for 24 h at 37°C with 5% CO_2_. TLR2 stimulation was assessed by measuring IL-8 levels in cell culture supernatants by enzyme-linked immunosorbent assay (ELISA) (Genscript).

### Assays of H. pylori Cag T4SS activity.

The ability of H. pylori to induce production of IL-8 when cocultured with gastric cells was determined as previously described (84). AGS gastric epithelial cells (ATCC CRL-1739) were grown in RPMI medium containing 25 mM HEPES and 10% FBS and were inoculated with H. pylori (multiplicity of infection [MOI] of 100) from liquid cultures that had been grown overnight. Cocultures were incubated for 4 h at 37°C with 5% CO_2_. Supernatant was collected, and IL-8 content was determined using an IL-8 ELISA (GenScript), according to the manufacturer’s specifications.

The ability of H. pylori to translocate CagA under conditions of coculture with gastric cells was determined as previously described (89, 90). H. pylori cells were cocultured with AGS cells for 7 h at 37°C with 5% CO_2_. Monolayers were washed in PBS and lysed in NP-40 lysis buffer containing Complete Mini protease inhibitors (Roche) and Phos-Stop phosphatase inhibitors (Roche). CagA translocation was assessed based on immunoblotting cell lysates with anti-CagA polyclonal antibody (Santa Cruz) and anti-phospho-tyrosine monoclonal antibody (pY-99; Santa Cruz).

### Immunoblotting.

Unless otherwise indicated, protein lysates were resolved by SDS-PAGE and proteins were transferred to nitrocellulose membranes. Membranes were blocked using PBS containing 0.1% Tween and 2% nonfat dry milk. Proteins were detected by incubating the membrane with primary antisera (diluted 1:5,000 to 1:10,000), followed by horseradish peroxidase-conjugated secondary antibody. Rabbit antiserum to H. pylori CagT and antiserum to HspB (a GroEL heat shock protein homolog) have been described previously (80, 91). Anti-CagT antiserum was preabsorbed to H. pylori BV199 (26695 Δ*rdxA* Δ*cagT*) prior to immunoblotting. Signals were detected by the use of an enhanced chemiluminescence (ECL) methodology.
