# Supplementary material for: Lipoprotein Processing and Sorting in Helicobacter pylori
Source: mBio. 2020 May 19;11(3):e00911-20. doi: 10.1128/mBio.00911-20 (PMC7240156; doi:10.1128/mBio.00911-20)
Supplement: FIG S1 [file mBio.00911-20-sf001.pdf]

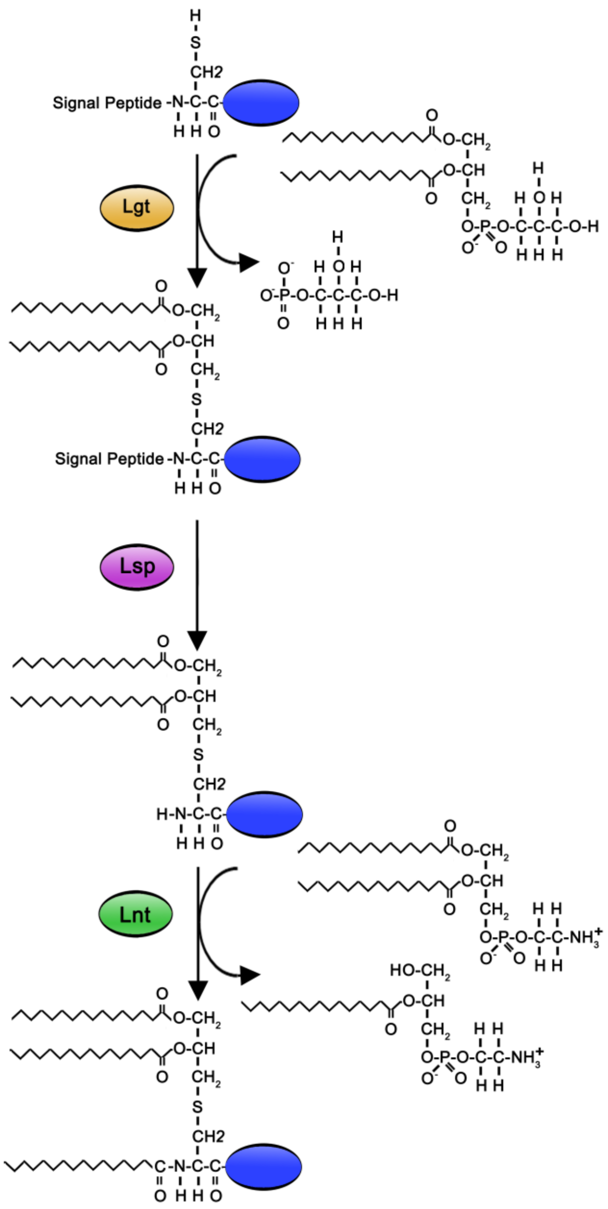

Supplemental Figure S1: Post-translational modifications in synthesis of lipoproteins in Gram-negative bacteria. The first modification is the addition of a diacylglyceride to the cysteine sulfhydryl of the preprolipoprotein, catalyzed by prolipoprotein diacylglyceryl transferase (Lgt). Amino acids preceding the cysteine are cleaved by prolipoprotein signal peptidase (Lsp), resulting in a diacylated apolipoprotein.

Finally, a fatty acid is ligated to the amino terminus of the amino-terminal cysteine by apolipoprotein N-acyltransferase (Lnt) to produce the mature triacylated lipoprotein.
